# What are you sexting? Parental practices, sexting attitudes and behaviors among Italian adolescents

**DOI:** 10.1186/s40359-020-00425-1

**Published:** 2020-06-15

**Authors:** E. Confalonieri, G. Cuccì, M. G. Olivari, M. Parise, E. Borroni, D. Villani

**Affiliations:** 1 Department of Psychology, CRIdee, Università Cattolica del Sacro Cuore, Milan, Italy; 2 Department of Psychology, Università Cattolica del Sacro Cuore, Milan, Italy; 3 Department of Psychology, Family Studies and Research University Centre, Università Cattolica del Sacro Cuore, Milan, Italy

**Keywords:** Parental practices, Parental monitoring, Attitude toward sexting, Sexting

## Abstract

**Background:**

Sexting has recently emerged as a public health and social issue. The present study had two aims: a) to preliminarily test adolescent gender differences on parental practices regarding adolescent online life, parental monitoring, adolescent attitude towards sexting and sexting behaviors; b) to separately test for male and female adolescents a conceptual model in which sexting behaviors are explained by the parental practices and monitoring, with the mediation of adolescent negative attitude towards sexting.

**Methods:**

Direct and indirect links between the variables in the model were investigated. The study was carried out with 541 participants. Participants were Italian adolescents (60% males; 40% females) aged 14 to 19 years (M_age_ = 16,19 years, SD_age_ = 1,31).

**Results:**

Results suggested that females sent more multimedia sexts, had a higher perception of risk associated with sexting and reported higher scores for both parental practices regarding adolescent online life and parental monitoring. Rules on Contents, Parental Knowledge, Adolescent Disclosure, and Parental Control resulted to be linked to both sexting attitudes and behaviors for male and female adolescents.

**Conclusions:**

Findings emphasize the important role that parents play in shaping attitudes and behaviors of both daughters and sons during adolescence.

## Background

Over the past 20 years, the progressive and increasingly rapid development of communication technologies has led to various changes in the way people, and especially adolescents, communicate, interact and relate to one another. As suggested by Weber and Dixon [65], the “digital culture” is progressively becoming more pervasive: adolescents and youth are the most digitally connected [72] using new communication media such as smartphones and social networks. These media allow them to be constantly in touch by sharing different types of contents, such as text messages, images and videos. Moreover, new technologies have also affected the way adolescents manage their intimate relationships, explore and express their sexuality [8].

Recently, sexting has emerged as a phenomenon attracting public health and social interest. Sexting is an English term combining the words “sex” and “texting” (message of text), originally referring to text messages containing sexual contents [26]. With the spread of new technologies, including smartphones and instant messaging and chat apps (e.g. WhatsApp, Snapchat, Telegram …), the term was also applied to the actions of posting on social networks and exchanging sexual contents, like pictures, videos or images [16, 26, 41]. Therefore sexting can be defined as the exchange (receiving, sending, forwarding and posting) of sexually explicit contents (texts and/or images/photos/videos of nude or semi-nude) on electronic media and the Internet [16, 17, 26, 33].

Recently, the literature has highlighted an increasing percentage of adolescents who practise sexting. According to one of the first international surveys carried out on sexting (Eurispes & Telefono [3, 4]), around 20% of adolescents received, privately sent, or posted sexts online, while more recent studies reported higher percentages between 60 and 80% [22, 42].

Research has investigated prevalence of sexting based on gender, but results are often inconsistent (for a review see: [17]). Some studies [42, 60, 67] showed higher frequency of sexting behaviors among male adolescents, while some other studies [9, 67, 71] suggested that boys receive sexts more frequently than girls and that the latter receive sext requests and send sexts more frequently. In order to better understand these gender differences inconsistency, it is possible to refer to the “Postfeminist” perspective (e.g. [46]) which focused on female sexual objectification in the contemporary media culture. According to this perspective, on the one hand, adolescent females are asked to produce a sexual content (i.e., the sext) as a form of self-display, which in some cases can be seen as a measure of attractiveness and a new form of feminine desirability. However, at the same time, those females who engage in such sexting behaviors are usually subject to peer moral condemnation and shaming. On the other hand, adolescent males could gain ratings showing or sharing girls’ pictures in their peer group [45].

In the extant literature, there are two main trends to interpret the phenomenon of sexting, reflecting two main perspectives. According to the developmental perspective [11, 12, 27, 36], sexting in adolescence may be considered as a normative expression of sexuality mediated by new technologies [36] and a new method that media-based communications have provided for facing developmental tasks related to adolescent sexuality [50], such as the expression, the exploration and the establishment of sexual identity. Following a second clinical perspective, sexting can be seen as a deviant behavior with severe consequences for health [19, 68, 70].

Sexting has sparked the concern of the adult world; parents, teachers, school administrators and the criminal justice system have begun to question themselves on this phenomenon (e.g., [1, 25]; Mattey [20]). Therefore, researchers have started to study sexting in order to identify relevant risk factors and protective factors and to promote adolescent ability to deal with this behavior and the related risks.

### Factors associated with sexting

Literature has identified individual and social factors linked to this phenomenon (for a review see: [17]).

Amongst individual factors, attitudes toward sexting play an important role. Researchers suggested that favourable attitudes towards sexting were positively associated with engaging in sexting behavior [33]. Walrave et al. [63] analysed attitudes towards sexting among Belgian secondary school students aged between 15 and 18 years, obtaining that the most accurate predictors of future sexting were higher perceived social pressure and positive attitudes towards this behavior. Research showed inconsistent results with regard to gender differences in attitudes towards sexting. Some studies reported that girls showed more negative attitudes toward sexting [54, 64], whereas a more recent study did not identify significant differences between females and males [35].

Amongst social factors, parents may play an important role. Although adolescence is a developmental period characterized by increased levels of autonomy, with increasingly important social relationships occurring and developing outside the family (i.e., peers and romantic partners), research on parenting suggested that parents continue to play a fundamental role in accompanying adolescent growth processes, even in relation to online behaviors, such as sexting [61]. Among parental practices that have been investigated in relation to online behaviors in general, research has mainly focused on parental mediation. Parental mediation processes deal with the way parents regulate and supervise their children’s media use [23]. Several studies have demonstrated that parental mediation influences children’s media use [48, 49, 56], helping reduce several online behaviors such as: Internet addiction [14, 30], cyberbullying [14, 32, 39], exposure to violent media content [15], contact with strangers [69] and online harassment [32].

More specifically, in their recent work on parents’ role in adolescents’ sexting behavior, Vanwesenbeeck et al. [61] described parental mediation strategies, distinguishing between restrictive and active mediation. The first strategy refers to parental attempts to control media access and to regulate the time that children spend with media [37, 57]. The second one regards parental efforts to actively explain media content to their children and convey their opinion, explaining and discussing the undesirable aspects of media content [58].

To our knowledge, only a limited number of studies have examined the relationship between sexting, parental practices and monitoring. This last is defined as parental awareness and supervision of children activities in several domains (e. g. friends, school and behavior at home), and parent-children communication [21]. Baumgartner et al. [5] carried out a 4-wave longitudinal study with 1762 Dutch adolescents aged 12–18 showing that adolescents who were more prone to online risk behavior, including sexting, were more likely to come from a less cohesive family, where each member knows little about the others and engages in their own activities without listening to other family members. Campbell and Park [13] carried out a telephone-based survey involving 800 adolescents aged 12–17 investigating whether children’s degree of control or autonomy over their technology use was associated with sexting. In this study, both parental monitoring and restrictive mediation resulted to be ineffective in preventing adolescent sexting, whereas a link between frequent communication with family members and lower percentages of sending and receiving sexual pictures emerged. West et al. [67] investigated the parental correlates of sexting among 949 Peruvian high school pupils aged 12–18. Results showed that for boys having parents setting rules about sending or receiving sexual messages was associated with decreased odds of sexting. Romo et al. [47] carried out a study involving 333 Hispanic adolescents aged 13–21 to evaluate the association between social media and sexual risk use, including sexting, and parental monitoring. They found that parental monitoring and parental discussion of privacy settings acted as protective factors for sexting, especially for females. In a study on 97 adolescents, Atwood et al. [2] examined the relationship between teenagers’ use of mobile Internet devices, involvement in potentially problematic digital behaviors, including sexting, parental mediation and parental attachment. Results showed that parental mediation did not affect sexting directly. However, adolescents who were strongly attached to their parents experienced less restrictive parental mediation and engaged in less risky online behaviors. Bianchi et al. [10] investigated associations between family functioning and sexting in a sample of 250 female adolescents aged 13–20. Authors distinguished between three different types of sexting: risky sexting behaviors (i.e., sharing sexts with many people), experimental sexting (i.e., exchanging sexts with a partner) and aggravated sexting (i.e., non-consensual forwarding of sexts). This study showed that sexting in general was negatively predicted by family communication. Risky sexting behaviors were positively predicted by age and negatively predicted by family communication, whereas experimental sexting was positively predicted by age and family flexibility, lastly the aggravated sexting was positively predicted by family enmeshment.

To our knowledge, there are no studies investigating the role of parental practices in shaping adolescent attitudes toward sexting. However, previous studies focusing on parents’ role in affecting adolescent sexual attitudes in ‘traditional’ settings (i.e., in the real world), have shown that parents and families have a protective function in determining adaptive teenagers’ sexual attitudes and behaviors [28].

In sum, the literature on the theme revealed, on a side, the presence of gender differences with regard to the frequency of sexting behaviors [9, 42, 60, 67, 71] and, on the other side, few or inconsistent evidence with respect to gender differences in the associations between parental practices and adolescent sexting attitudes and behaviors [47, 67]. Therefore, we believe that the investigation of these associations distinguishing between males and females deserves more attention. This could help clarify the literature evidence and understand better whether and how different parental practices may be linked to attitudes and behaviors in males and females.

### The present study

The present study had two aims. The first one was to preliminarily test adolescent gender differences about parental practices regarding adolescent online life (i.e., Parental Active Mediation, Rules on Time, Rules on Contents, Quality and Frequency of Communication) and parental monitoring (i.e., Parental Knowledge, Adolescent Disclosure, Parental Control), adolescent attitude towards sexting (i.e., adolescent risk perception of sexting) and sexting behaviors (i.e., sending sexual explicit text messages and sending sexual explicit image and/or video). The second one was to test a conceptual model for male and female adolescents separately, in which sexting behaviors (i.e., sending sexual explicit text messages and sending sexual explicit image and/or video) are explained by the parental practices regarding adolescent online life (i.e., Parental Active Mediation, Rules on Time, Rules on Contents, Quality and Frequency of Communication) and parental monitoring (i.e., Parental Knowledge, Adolescent Disclosure, Parental Control), through the mediation of adolescent negative attitude towards sexting (i.e., adolescent risk perception of sexting). In the model, we investigated both direct and indirect links between the variables.

Drawing on the available evidence, we expected that parental practices regarding adolescent online life and parental monitoring would be negatively linked to adolescent’s engagement in sexting [2, 13, 47, 67]. Moreover, we expected a positive link between parental practices regarding adolescent online life and parental monitoring with adolescent negative attitude toward sexting [28] which in turn would be negatively linked to adolescent engagement in sexting [33, 63].

According to the few evidences regarding gender differences in relation to parental practices and adolescent sexting behaviors, we expect that for females parental active mediation, quality and frequency of communication and monitoring would be positively linked to adolescent negative attitude toward sexting which in turn would be negatively linked to adolescent engagement in sexting. Moreover, we expect that these parental practices would be negatively linked to adolescent engagement in sexting [47]. For boys, we expect that parental Internet restriction would be positively linked to adolescent negative attitude toward sexting, which in turn would be negatively linked to adolescent engagement in sexting. Moreover, we expect that these parental practices would be negatively linked to adolescent engagement in sexting [67].

## Method

### Participants and procedure

An a priori power analysis was conducted using G*Power3 [24] to test the desired sample size for the path analysis with an alpha of .05. to achieve a power of .95. A minimum sample of 138 participants was required.

Participants were selected through a convenience sample from northern Italian high schools. The research was presented to headmasters who offered the participation of the school voluntarily and identified the classes to be involved in the research. A total of 10 schools participated in the research. Once the headmaster had granted the permission, class teachers allowed the administration of the study during their classes. Students’ parents received a letter presenting the study and both parents were asked to provide their written consent. Adolescents aged 18 or older signed the written consent to participate in the research.

Data were collected between September and November 2019. The questionnaires were administered online by a researcher during classes in a room equipped with personal computers.

Originally, 600 high school students were involved to participate in the research. Among these, 557 accepted to take part in the research by returning the signed consent form. Subsequently, data from 16 adolescents were excluded from the analyses because they had not fully completed the questionnaires about parental practices or sexting behaviors.

Thus, participants were 541 Italian adolescents (60% males; 40% females) aged 14 to 19 years (M_age_ = 16,19 years, SD_age_ = 1,31), living in the North of Italy.

At the time at which of the study was conducted, 16% of participants had a romantic relationship, 19% was dating someone and 65% was single. The 28% of the sample reported having already had a sexual intercourse.

The approval for the study was obtained from the Ethical Commission of the Department of Psychology of Università Cattolica del Sacro Cuore of Milan.

### Instruments

We asked participants to complete an online questionnaire in about 45 min investigating the following constructs.

#### Socio-demographic characteristics

Participants firstly completed items on socio-demographic variables regarding gender, age, dating and sexual experience.

#### Attitude toward sexting

Participants completed one subscale of Sexting Attitude Scale [66], a 19-item scale assessing attitude toward sexting. We employed the Perceived Risk subscale (5 items, i.e., *Sending sexually racy pictures leaves me vulnerable*) to evaluate adolescent negative and risky perceptions towards sexting. Responses were given using a five-point Likert scale ranging from “Absolutely not true” (1) to “Absolutely true” (5).

Parental practices regarding adolescent online life were measured focusing on: Parental Active Mediation, Parental Internet Restriction, Frequency and Quality of Communication.

#### Parental active mediation

Participants responded to a three-item scale that was originally used by Atwood et al. [2] to measure participants’ perceptions of parental active mediation (i.e., *“How frequently in the past six months a parent has talked to you about what is appropriate and inappropriate to view on the Internet and mobile devices?”*). Responses were given using a five-point Likert scale ranging from “Never” (1) to “Always” (5).

#### Parental internet restriction

To assess parental Internet restriction participants were administered two measures: (a) Rules with regard to time spent on the Internet [6, 59], a six-item scale assessing participants’ perceptions of parental rules on the time spent in Internet (i.e., *“My parents allow me to go on the internet as often as I want to”*). Responses were given using a five-point Likert scale ranging from “Never” (1) to “Always” (5). (b) Rules with regard to content of Internet use [6, 59], a three-item scale assessing participants’ perceptions of parental rules on the contents searched in Internet (i.e., *“My parents allow me to have online contact with anyone”*). Responses were given using a five-point Likert scale ranging from “Absolutely not true” (1) to “Absolutely true” (5).

#### Frequency and quality of communication

Participants responded respectively to the three-item Frequency of communication regarding Internet use scale and the three-item Quality of communication regarding Internet use scale [6, 59] which measure participants’ perceptions of the frequency and quality of communication with parents regarding Internet use (respectively, i.e., *“How often do you and your parents talk about what you are doing on the internet?”; “When my parents and I talk about my internet use, I feel comfortable”*). Responses were given using a five-point Likert scale ranging from “Never” (1) to “Very often” (5).

#### Parental monitoring

Participants completed three subscales of the Parental Monitoring Questionnaire [40, 53], a 25-item scale assessing parental monitoring. The three subscales were: (a) Parental Knowledge (9 items, i.e., *“Do your parents: know what you do during your free time?”*), to evaluate adolescent perceptions of parental knowledge about one’s whereabouts, activities and peers; (b) Adolescent Disclosure (5 items, i.e., *“Do you hide a lot from your parents about what you do during the day?”*), to evaluate adolescents’ tendency to provide unsolicited information; (c) Parental Control (6 items, i.e., *“Do you need to have your parents’ permission to stay out late on a weekday evening?”*), to evaluate whether the adolescent is required to inform parents about where he or she will be and with whom. Responses were given using a five-point Likert scale ranging from “Never” (1) to “Always” (5).

#### Sexting behaviors

Participants completed two ad hoc items especially designed for this study to assess their engagement in sexting behaviors. We focused on two behaviors: sending sexual explicit text messages (*“Have you ever sent a sexually explicit text message to anybody*?”) and sending sexual explicit images and/or videos (*“Have you ever sent your own sexy photos or videos where you are partially or completely naked to anybody?”*). The choice to distinguish between the two types of content (i.e., text messages and images and/or video) is since the literature on the theme is not always clear and uniform when speaking about sexting behaviors. Studies have indeed investigated more in general “sending sexts” or just specific kind of sexts such as “explicit sexual images” (for a review see [17]). Responses were given using a five-point Likert scale ranging from “Never” (1) to “Always” (5). Prior to answering these two items, the following information appeared on the screen to make sure adolescents understood the meaning of the questions: *“When you will read the term SEXUALLY EXPLICIT TEXT MESSAGES we refer only to text messages with sexual contents written by you; When you will read the term SEXY PHOTOS OR VIDEOS we refer only to images or videos with sexual contents of you”*.

### Data analysis

**Fig. 1:**
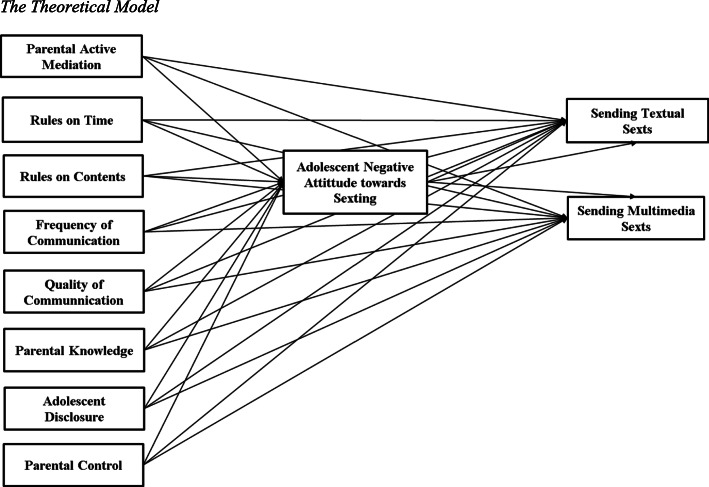
The Theoretical Model

A path-analysis model with a mediation was tested using Amos Graphics 21, analyzing both direct and indirect links. In the theoretical model (see Fig. 1), parental practices regarding adolescent online life and parental monitoring were expected to explain the two sexting behaviors, through the mediation of adolescent negative attitude towards sexting.

We tested the model separately for adolescent gender. Therefore, two models were performed.

We started from a saturated model (see Fig. 1) with all the direct and indirect links and we proceeded with a step-by-step procedure by removing from the model all non-significant links among variables. Modification indexes were used to identify other direct and indirect links and correlations that were not previously considered. Each model was computed using Maximum Likelihood estimation method to explore the theoretical model hypothesized. At each step of the model, we examined Goodness-of-fit indexes: Chi square test, RMSEA, and CFI. Models with acceptable fit presented non-significant Chi square value (*p* > 0.01 since the large sample), RMSEA < .08, CFI > .90 [7]. Significance of indirect paths was estimated performing Bootstrap (Percentile Confidence Intervals type).

The final version of each model resulted by the correlations and the estimated paths. As such, the final version only contains only significant links and the evaluation of the modification indexes.

The Results paragraph reports the final version of each model, showing only the significant links between the variables.

## Results

### Preliminary analyses on gender differences

Independent sample t-tests were conducted to examine gender differences (Table 1) on adolescent negative attitude towards sexting, sexting behaviors, parental practices regarding adolescent online life and parental monitoring. As showed in Table 1, females reported higher scores than males for both negative attitude towards sexting and sending multimedia sexts. No gender differences emerged for sending textual sexts. With regard to parental practices about adolescent online life and parental monitoring, females reported higher scores than males for all the variables, except for Rules on Time where no differences emerged.

**Table 1:** Cronbach’s alphas, Independent Sample T-Test results comparing male and female adolescents for adolescent risk perception of sexting, parental practices regarding adolescent on-line life, parental monitoring and sexting behaviors

			Males		Females		
		Cronbach’s alpha	M	SD	M	SD	*t* test
Negative attitude towards Sexting	Adolescent Risk Perception of Sexting	.82	3.74	0.95	3.91	0.92	−2.065^*^
Parental practices regarding adolescent on-line life	Parental Active Mediation	.76	1.96	0.84	2.22	1.05	−3.100^**^
	Frequency of Communication	.73	1.97	0.83	2.29	0.93	−4.140^***^
	Quality of Communnication	.81	3.02	1.08	3.31	1.00	−3.149^**^
	Rules on Time	.82	2.12	0.85	1.98	0.80	1.836
	Rules on Contents	.83	2.36	1.13	2.63	1.18	−2.753^**^
Parental Monitoring	Parental Knowledge	.75	3.53	0.59	3.77	0.57	−4.639^***^
	Adolescent Disclosure	.78	3.08	0.82	3.50	0.83	−5.812^***^
	Parental Control	.83	3.35	0.98	3.67	0.91	−3.848^***^
Sexting Behaviors	Sending Textual Sexts	/	1.74	1.05	1.73	1.01	.094
	Sending Multimedia Sexts	/	1.27	0.63	1.47	0.83	−.2.947^**^

p*< .05; p**< .01; p***≤ .001

Considering that gender differences emerged, the preliminary results supported the choice to test two different models based on adolescent gender.

### Primary analysis

We ran two path-analysis models, in which parental practices regarding adolescent online life (i.e., Parental Active Mediation, Rules on Time, Rules on Contents, Quality and Frequency of Communication) and parental monitoring (i.e., Parental Knowledge, Adolescent Disclosure, Parental Control) explained adolescent sexting behaviors in terms of sending sexual explicit test messages (labeled: “Sending Textual Sexts”) and sending sexual explicit image and/or video (labeled: “Sending Multimedia Sexts”) through the mediation of Adolescent Negative Attitude towards Sexting.

The models were tested separately for male and female adolescents, testing the statistical significance of direct and indirect paths. Only significant paths are reported in figures.

#### Models for gender

**Fig. 2:**
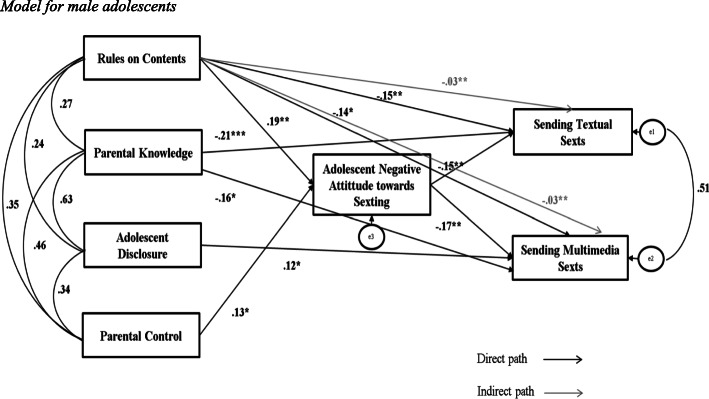
Model for male adolescents. p*< .05; p**< .01; p***≤ .001

In the male sample (*N* = 325), Parental Control and Rules on Contents were found to directly explain Adolescent Negative Attitude towards Sexting. Rules on contents also directly explained Sending both Textual and Multimedia Sexts. Moreover, Disclosure resulted to directly explain Sending Multimedia Sexts as well as Parental Knowledge that also directly explained Sending Textual Sexts. Adolescent Negative Attitude towards Sexting in turn directly explained Sending both Textual and Multimedia Sexts. Finally, Rules on Contents resulted to indirectly explain Sending both Textual and Multimedia Sexts through the mediation of Adolescent Negative Attitude towards Sexting. No other indirect paths resulted to be significant (see Fig. 2). The model presented acceptable Goodness-of-fit indices: χ^2^_(5)_ = 5.807 (*p* = .326), CFI = 0.99, RMSEA = .02.

**Fig. 3:**
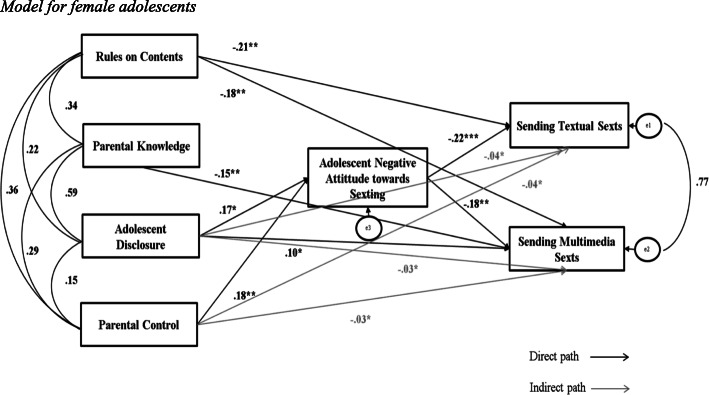
Model for female adolescents. p*< .05; p**< .01; p***≤ .001

In the female sample (*N* = 216), Parental Control and Disclosure were found to directly explain Adolescent Negative Attitude towards Sexting. Disclosure also directly explained Sending Multimedia Sexts. Rules on Contents was found to directly explain both Sending Textual and Multimedia Sexts and Parental Knowledge to directly explain Sending Multimedia Sexts. Adolescent Negative Attitude towards Sexting in turn directly explained Sending both Textual and Multimedia Sexts. Lastly, Parental Control and Disclosure indirectly explained Sending both Textual and Multimedia Sexts through the mediation Adolescent Negative Attitude towards Sexting (see Fig. 3). The model presented acceptable Goodness-of-fit indices: χ^2^_(6)_ = 12.106 (*p* = .060), CFI = 0.98, RMSEA = .07.

## Discussion

Our study aimed at testing a conceptual model in which parental practices regarding adolescent online life and parental monitoring explained adolescent sexting behaviors through the mediation of adolescent negative attitude towards sexting.

Consistent with previous evidence [9, 67, 71], our preliminary results on gender differences regarding sexting frequency among adolescents showed that females sent more multimedia sexts but also had a higher perception of risks associated with sexting if compared to males. Although these two results may appear to be in contrast, they are in line with the literature showing that despite a higher awareness concerning sexting consequences and a more negative attitude towards sexting, female adolescents are those who practice more sexting [62]. It is possible that for females some other factors associated with motivation for sexting (e.g. peer or partner pressure, impress someone they like, blackmail) impact on the choice to engage in sexting behaviors. These results may be better understood in light of a “Postfeminist” perspective, which suggests that girls engaging in sexting are subject to a sexual double standard: they are asked to send sexts as a form of self-display but at the same time they become object of moral condemnation by the peer group [45]. This last aspect may help explain why girls showed a negative attitude towards sexting but also sent more multimedia sexts.

Preliminary results highlighted some gender differences as well: females scored higher on all the variables investigating both parental practices regarding their online life (except for Rules on Contents) and parental monitoring. This result is in line with previous findings: empirical studies, in fact, showed that females perceived to be more monitored in their online activities [47] and reported more parental knowledge, control and adolescent disclosure than males in off-line contexts [18, 29, 31, 53]. It could be that parents tend to control more carefully and be more aware of their daughters’ lives since they perceive them as being more exposed and vulnerable to several risks both in online and off-line activities and also because females are more willing to self-disclose to their parents [34].

As far as our main aim is concerned, the model was tested separately for male and female adolescents and interesting results emerged.

First, as expected, in line with previous studies [33, 63] a negative attitude towards sexting was found to be associated with less engagement in sexting behaviors for both males and females. Therefore, adolescents who are more aware about the risks connected to sexting are less prone to send both text and multimedia sexts.

Another general consideration is that the two final models include the same parental variables: Rules on Contents, Parental Knowledge, Adolescent Disclosure and Parental Control. Parental practices, as perceived by the adolescents, are important in shaping sexting attitudes and behaviors in adolescence rather than a specific parental behavior. Beside discipline (i.e., Rules on Contents and Parental Control), what emerges as especially relevant is the possibility of a dialogue and an open communication between children and their parents (i.e., Parental Knowledge, Adolescent Disclosure) regarding everyday life and activities. It seems important that parents show interest for their children’s lives, giving value to both the experiences they have with peers in the virtual and real world and that adolescents disclose information regarding their personal and social lives with parents. It is possible that a climate characterized by sharing and closeness also favors the negotiation of rules with children, and this, in turn, could promote their internalization. This process is typical of autonomy supportive parenting [57], as well as of authoritative parenting [44], in which rules are discussed and shared with children, stimulating their critical thinking and problem solving and providing them an opportunity of growth. Empirical studies focusing on parenting styles have indeed demonstrated immediate and long-term protective effects of the authoritative style for adolescent development [43, 55].

More in depth, an interesting result is that among parental practices regarding adolescent online life, only Rules on Content resulted to directly (and indirectly only for females) explain both adolescent sexting attitudes and behaviors. Rules on Contents provided by parents contributed to shape negative attitudes towards sexting and to decrease adolescent engagement in sexting behaviors. This result, albeit unexpected, highlights the importance for parents to control and provide rules on the contents to which the adolescent may have access rather than on the time spent online. Therefore, it is important for parents to negotiate clear rules concerning the allowed online contents and type of Internet activities (e.g. chatting with friends and/or unknown people) since this parental behavior resulted to have an influence on both sexting attitudes and behaviors. It is possible that by providing these clear rules parents also promote in the adolescent a clearer vision of the risks connected to on line behaviors. Thus, it is possible to hypothesize that this could lead the adolescents to develop their own perception of these risks thanks to the process of internalization of parental norms and values [51]. These processes could guide and promote adolescent’s healthy behavior in future online life.

As for parental monitoring, Parental Knowledge, Parental Control and Adolescent Disclosure resulted to be significantly linked to adolescent sexting attitudes and behaviors in the expected direction. The first practice, Parental Knowledge, for both males and females resulted to directly explain sexting behaviors. Therefore, parental awareness about adolescent’s whereabouts, activities and peers may decrease adolescent engagement in sexting behaviors. This result is in line with the literature that has in general already suggested that Parental knowledge of adolescents’ online experiences could lead to a safer use of Internet and positively affect children behaviors [61]. However, it appears that Parental Knowledge does not increase or diminish the awareness concerning risks about sexting. On the contrary, Parental Control for both males and females resulted to be directly linked only with adolescent negative attitude towards sexting. For females, Parental Control, despite weakly, resulted also to be indirectly linked to adolescents sexting behaviors through the mediation of adolescent negative attitude towards sexting. Therefore, the fact that the adolescent should inform parents about where and with whom he/she will be, appears to be an important factor in shaping adolescents’ way of thinking and feeling about sexting. This result may seem surprising, since the literature has widely demonstrated that, in general, a controlling parenting style has a negative effect on children’s and adolescents’ development [52]. We believe that this result, together with the previous one regarding the importance of setting clear rules in terms of allowed and forbidden online contents and activities, should be read with caution. We may hypothesize, in fact, that these restrictive parental behaviors can be protective if and when they are combined with different parental practices, such as the promotion of an open communication with children regarding their life (and not only the virtual one). The ability to balance communication and control can be ascribed to authoritative parental practices, that the literature has demonstrated to widely and positively impact on adolescent development [43, 55].

The last engaging result concerning parental monitoring tackles Adolescents Disclosure, which, unexpectedly, resulted to be linked with higher engagement in sending multimedia sexts for both males and females. Moreover, for females Adolescents Disclosure resulted to be indirectly linked, despite weakly, to adolescents sexting behaviors through the mediation of adolescent negative attitude towards sexting. Our finding is not in line with previous evidence, which found that child self-disclosure was linked to lower engagement in anti-social and deviant behavior, also in the online context (i.e., adolescent online aggression). In particular, the child’s willingness to self-disclose information resulted to protect more children from anti-social behavior than parental solicitation and control [38, 53]. In our opinion, a possible explanation for the positive association between Adolescents Disclosure and the sexting behavior can be that if adolescents spontaneously share information and talk with their parents about their social life, it is possible that they will talk about their romantic relationships and also about sending sexy messages or images to the romantic partner. This aspect requires further consideration in future research. From this point of view, the sexting behavior will assume the normative function of an expression of sexuality mediated by new technologies proposed by the developmental perspective [36], and could not be necessarily a deviant or risky behavior. However, in this study we did not investigate the relational context where sexting takes place, therefore the latter remains a hypothesis warranting further investigation in the future.

Findings of the present study resulted only partially in line with our main hypothesis. As far as females are concerned, we did not find a significant role played by active mediation, quality and frequency of communication, while, for boys, only the provision of rules on contents (as a practice of Parental Internet Restriction) resulted to be associated with adolescent sexting. Importantly, the findings of the present study suggest that the practices linked with parental monitoring are associated with sexting attitudes and behaviors in adolescence more than the ones regarding adolescent online life. We believe that this finding can be explained by the fact that sexting is not just a private phenomenon enacted by adolescents only online (e.g. such as pornography). Indeed, sexting implies adolescents’ social life since it is a relational behavior that involves at least two people. For this reason, we believe that parental control, knowledge about adolescents’ life and adolescent’s disclosure, which foster parent-child communication, play an important role in making adolescents more aware about risks in general and more responsible about sexting behaviors. This is true for both male and female adolescents.

The present study has some limitations that future research should address. First, this is a cross-sectional study so a longitudinal design could help verifying causal links among variables. Secondly, since we did not investigate the reasons why a sext is sent nor the recipient of the sexts, it is not possible to distinguish between normative and non-normative sexting. Future studies should also include the investigation of the context and the conditions where sexting takes place (e.g., romantic relationships, peer group, induced by blackmail etc..). By focusing on the context, indeed, it will be interesting to further understand how the process of gender socialization and the masculine and feminine relational tasks about sexual and romantic relationships may be linked to sexting among male and female adolescents. Moreover, in the future it would be interesting to investigate age differences and explore whether there are differences in how parents may influence sexting behaviors and attitudes of early and late adolescents.

Beyond the limits, our findings emphasize the important role that parents still play in adolescence in shaping attitudes and behaviors of both daughters and sons. On the one hand, the innovative attempt of the present study is to clarify literature findings on the associations between adolescent sexting and parental practices which resulted to be inconsistent. On the other hand, to our knowledge, the present study is the first one to investigate at the same time parental practices regarding adolescent online life but also parental monitoring which includes parental practices regarding adolescent daily life “off-line”.

## Conclusion

We believe that our study could provide a theoretical base for the implementation of intervention programs on sexting addressed to both adolescents and their parents. As for the adolescents, they should be informed and made aware of the risks and consequences of sexting. This is particularly true for females who resulted to send more multimedia sexts than males. Parents should be aware of the importance they could play in educating their children about sexting. In particular, by providing clear rules, parents will help their children to gain an internalization of rules regarding sexting. Moreover, given the important role played by parental knowledge, parents should learn how and be encouraged to communicate with their sons and daughters encouraging them to speak openly about sexual behaviors, and to listen to adolescents’ opinions in a nonjudgmental way.

## Data Availability

All data generated or analyzed during this study during the current study are not publicly available due to ethical reasons.
